# An internet-based intervention for people with psychosis (EviBaS): study protocol for a randomized controlled trial

**DOI:** 10.1186/s12888-018-1644-8

**Published:** 2018-04-13

**Authors:** Nina Rüegg, Steffen Moritz, Thomas Berger, Thies Lüdtke, Stefan Westermann

**Affiliations:** 1 Department of Clinical Psychology and Psychotherapy, University of Bern, Fabrikstrasse 8, 3012 Bern, Switzerland; 2 Department of Psychiatry and Psychotherapy, University Medical Center Hamburg-Eppendorf, Hamburg, Germany; 3 Department of Psychology, Faculty of Health Sciences, UiT - The Arctic University of Norway, Tromsø, Norway

**Keywords:** Online intervention, CBT, Psychosis, Schizophrenia, Internet, Guided self-help

## Abstract

**Background:**

Evidence shows that internet-based self-help interventions are effective in reducing symptoms for a wide range of mental disorders. To date, online interventions treating psychotic disorders have been scarce, even though psychosis is among the most burdensome disorders worldwide. Furthermore, the implementation of cognitive-behavioral therapy (CBT) for psychosis in routine health care is challenging. Internet-based interventions could narrow this treatment gap. Thus, a comprehensive CBT-based online self-help intervention for people with psychosis has been developed. The aim of this study is the evaluation of the feasibility and efficacy of the intervention compared with a waiting list control group.

**Methods:**

The intervention includes modules on delusion, voice hearing, social competence, mindfulness, and seven other domains. Participants are guided through the program by a personal moderator. Usage can be amended by an optional smartphone app. In this randomized controlled trial, participants are allocated to a waiting list or an intervention of eight weeks. Change in positive psychotic symptoms of both groups will be compared (primary outcome) and predictors of treatment effects will be assessed.

**Discussion:**

To our knowledge, this project is one of the first large-scale investigations of an internet-based intervention for people with psychosis. It may thus be a further step to broaden treatment options for people suffering from this disorder.

**Trial registration:**

NCT02974400 (clinicaltrials.gov), date of registration: November 28th 2016.

**Electronic supplementary material:**

The online version of this article (10.1186/s12888-018-1644-8) contains supplementary material, which is available to authorized users.

## Background

Schizophrenia and other psychotic disorders are severe mental disorders with heterogeneous symptom profiles encompassing positive symptoms such as persecutory delusions and auditory verbal hallucinations as well as negative symptoms such as social isolation and avolition [1]. In addition, they are accompanied by neuropsychological impairments in attention, memory, and executive functioning [2–6]. Sleep is impaired in the majority of people experiencing persecutory delusions [7] and levels of worrying are high [8, 9]. Besides symptoms, stigmatization is a major source of distress in people diagnosed with schizophrenia [10], even in the context of mental health care [11]. Lifetime prevalence of schizophrenia is about 1% and stable across different regions of the world and cultures [12]. Schizophrenia is accompanied by an enormous individual and societal burden [13, 14] and lies on position eight of the leading causes of disability-adjusted life years in 15- to 44-year-olds [15]. About 65% of individuals with a first episode relapse during the subsequent three years [16], resulting in inpatient costs about two to five times higher compared to non-relapsed patients [17].

As a complementary or alternative treatment option to antipsychotic medication [18], cognitive behavioral therapy for psychosis (CBTp) has emerged as an evidence-based treatment option for patients with schizophrenia and related disorders [19–24]. CBTp targets psychological mechanisms of symptom formation and maintenance that were primarily identified or corroborated using experimental psychopathology research [25–27]. The therapeutic framework and techniques of CBTp are to a large extent similar to those of cognitive behavioral therapy (CBT) for depressive or anxiety disorders (cognitive restructuring, reality testing, etc.). For example, the distress (consequence) related to hearing voices (situation) is assumed to be determined not by hearing voices per se, but predominantly by automatic thoughts and the according belief system. Consequently, alternative helpful beliefs about voices established with the help of cognitive techniques are supposed to result in less distress [28]. CBTp is likely to be effective for patients who choose not to take antipsychotic medication, too [29]. In regular mental health care, the effectiveness of CBTp has also been asserted [30], and neurocognitive deficits, comorbidity and poorer functioning pose no barrier to improvement during CBTp [28]. Consequently, national regulations such as the United Kingdom National Institute for Health and Care Excellence (NICE) guideline recommend that CBTp should be offered to every person with psychotic symptoms [31].

Acceptance and Commitment Therapy (ACT) focuses on noticing rather than changing thoughts and feelings [32]. ACT seems to be effective in treating mental health problems [33]. In schizophrenia, ACT helps people to cope with psychotic experiences using strategies such as cognitive distancing, which is characterized by learning to see one’s belief as a hypothetical statement rather than a fact. Instead of trying to change, modify, or control odd cognitions or disturbing sensory states, patients are encouraged to instead simply be aware of these experiences [34]. A meta-analysis showed a medium-sized effect of ACT on symptoms of psychosis [35].

The third type of treatment is the Metacognitive Training for psychosis (MCT), developed specifically for people with schizophrenia [36, 37]. MCT invites participants to critically evaluate cognitive biases such as jumping to conclusions and overconfidence in their thinking (metacognition). These biases might increase the likelihood of psychotic symptoms [38]. Studies show that MCT is efficacious in reducing psychotic symptoms [39, 40].

Despite the availability of evidence-based treatments for schizophrenia, 69% of patients remain untreated in countries with low and middle income [41]. In particular, the need for psychosocial treatments including CBTp remains unmet [42]. Even in highly developed countries such as the United Kingdom or Germany, the treatment gap for schizophrenia is large. In theory, the NICE guidelines proclaim that CBTp is mandatory for the treatment of psychosis [31]. In practice, more than 50% do not receive even a single session of CBTp [43]. In Germany, CBTp is virtually not represented in the mental health service [44]. To sum up, CBTp is effective, recommended, and has great potential to alleviate psychological distress, but only a small fraction of patients with psychosis receives CBTp.

Internet-based cognitive behavioral therapy (iCBT) can help to overcome treatment gaps in many mental disorders [45]. In several psychological disorders, including anxiety and depression, internet-based treatments have proven to be efficacious and effective in randomized controlled trials (RCTs; for a comprehensive review, see [46]). Most of the growing body of evidence comes from studies evaluating guided internet-based self-help treatments. While patients work their way through a structured self-help program that is typically based on CBT manuals, therapists or coaches assist and support them via a secured e-mail system. Meta-analyses on internet-based treatments show a superiority of guided interventions in comparison to unguided, automated programs in terms of efficacy, adherence to treatment, and drop-out rates [47–49]. Main advantages of guided internet-based treatments include: (1) low-threshold accessibility, (2) flexible usage independent of time and place at a self-determined pace, (3) high levels of anonymity and privacy (which is an attractive feature for many persons with a mental disorder due to their fear of stigmatization) and (4) low costs of delivery to large populations [50].

People diagnosed with schizophrenia use the internet [51] and are able and willing to use mental health services on the internet, such as peer-to-peer support [52]. The feasibility of internet-based treatments for people with psychosis (iCBTp) is well documented for web-based interventions [53, 54] and also reported for smartphone interventions [55]. However, current internet-based programs differ in their comprehensiveness and focus. For instance, mixed results have been reported regarding the efficacy of internet-based psychoeducation programs [56], and the efficiency of internet-based programs targeting medication management [53, 57]. There is a pilot study on a more comprehensive web-based, CBTp-oriented program for auditory verbal hallucinations, but this program was delivered via computers in mental health care centres (and not online). The study provided promising results using an uncontrolled pre-post-design (Cohen’s d = 0.58) [58]. None of the 21 participants with schizophrenia reported that the program was unhelpful and the authors report no adverse events, highlighting the feasibility of iCBTp in a computerized self-help format. A recent investigation of aforementioned program in an RCT design showed a comparable effect of the web-based intervention and usual care on levels of auditory hallucinations [59]. The study was able to show that patients with schizophrenia who used the web-based program, however, had increased significantly in social functioning and their knowledge about CBTp was larger than of those who did not use the program. In another study that investigated iCBT for people with schizophrenia, the web-based program specifically targeted comorbid depressive symptoms. The intervention lead to a significant decline in depression severity [60].

In summary, there is preliminary evidence that iCBTp for people diagnosed with schizophrenia could be beneficial. However, to the best of our knowledge, no larger trials on comprehensive treatments have been conducted. The overarching goal of this RCT is to evaluate a guided internet-based self-help intervention for people with psychosis. We developed a web-based program that is comprehensive in many respects: The program is not only based on CBT but also includes elements from its third wave, specifically ACT and MCT [34, 36]. Schizophrenia patients often have comorbidities, such as depression, which should be addressed in an appropriate treatment [61]. This program offers additional interventions for such comorbidities. Disrupted sleep and worrying, among other secondary symptoms, are crucial in the formation and maintenance of psychotic disorders [25]. These factors are considered in the intervention as well. According to a review, the effects of smartphone-enhanced self-help are promising [62]. The intervention therefore includes an accompanying smartphone app for access in symptom-relevant situations in daily life. The app is expected to facilitate a transfer of skills to real world settings. Finally, a specific goal of the intervention was not to overstate negative consequences of the disorder [63] and solely focus on deficits, but to specifically target resources of the participants [64].

Treatment adherence in schizophrenia has been a well discussed topic predominantly in medication treatment [65]. But also in psychological treatments, rather high dropout rates are reported (e.g. prematurely terminated treatments by 45% of patients) [66]. This led us to look for factors that might influence treatment adherence. Among others, suggested mediators are treatment motivation [67] and working alliance with the therapist [68]. Overall, the study tests whether a comprehensive internet-based self-help program with an accompanying smartphone app reduces symptomatology in people with schizophrenia.

## Methods

### Study design

**Fig. 1:**
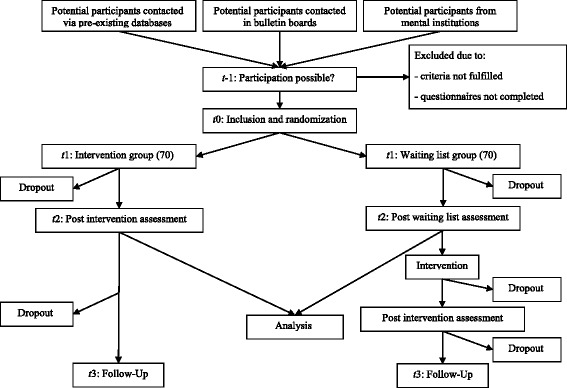
Participant flow

The study is an RCT of parallel design comparing the efficacy of guided internet-based self-help treatment for patients with schizophrenia to a waiting list control group (Fig. 1). Participants in the control group receive access to treatment after the intervention period of eight weeks. The long-term effect of the intervention is measured by a follow-up assessment six months after the intervention period has ended and is not part of the RCT design.

### Sample size

A power analysis with the software G*Power [69] resulted in a target sample size of 128 to detect a medium-sized effect (*f* = 0.25) with α = 0.05 and a power of 0.80 for an ANCOVA. Including an assumed attrition rate of about 10%, the final number of participants should reach 140.

### Recruitment

Participants will be recruited in three different ways: First, the study staff will contact former patients with diagnoses of schizophrenia who consented to get contacted and inquire whether they are interested in participating in this study via e-mail. Second, study information will be sent out to psychiatric institutions in Switzerland and Germany. The health staff at those institutions can then distribute the information to interested and suitable patients directly, for example those patients who leave the institution and look for a continuation treatment. Lastly, online bulletin boards and informative websites on psychoeducation specifically created for people affected by schizophrenia will be targeted for publishing descriptions and links to the study.

### Eligibility criteria

To be included, participants must fulfill all inclusion criteria and not show any exclusion criteria described below. Suffering from other psychiatric disorders such as depression or anxiety disorders (part of the former ‘axis I’ disorders) does not lead to exclusion as long as the schizophrenia spectrum disorder is the primary diagnosis.

Inclusion criteria are:

1. An age of 18 years or older.
2. Provision of electronic informed consent.
3. Access to the internet.
4. Command of the German language.
5. Fulfilling the diagnostic criteria for either schizophrenia, delusional disorder or schizoaffective disorder in their lifetime according to the structured Mini International Neuropsychiatric Interview (MINI) [70] administered during a telephone interview. These diagnoses are allowed to be partly remitted.
6. A score of three or higher on the items assessing delusions (P1), hallucinations (P3) or suspiciousness/persecution (P6) on the Positive and Negative Syndrome Scale (PANSS) [71] showing that some positive symptoms remain.
7. Simultaneous treatment with antipsychotic medication (or regular psychological or psychiatric care in Germany).

Exclusion criteria are:

1. Acute suicidality.
2. Representing an acute danger for others.
3. No agreement on compiling an emergency plan.
4. Diagnosis of an acute neurological disease of the central nervous system that needs to be treated.

### Randomization

Participants eligible for inclusion will be randomly allocated to one of the two groups (intervention or waiting list control group). Randomization and allocation will be prepared in advance by an independent researcher. This researcher will remain blinded to all processes within the intervention. An automated, web-based randomization service (www.random.org) [72] will be used to generate the randomization list. The allocation ratio will be 1:1.

### Intervention

The intervention called *EviBaS* (for ***Evi****dence-****Ba****sed ****S****elf-help* intervention) consists of an online program based on CBT principles, while also including components of ACT and MCT treatments. A smartphone app provides the possibility of exercising the modules in everyday life. There are 11 text-based modules in the online program, addressing a variety of topics (see Table 1). Each module includes texts and a worksheet. The worksheets can also be accessed via the optional app. The only mandatory module is the introductory one. After completion, the participants can choose from the remaining ten modules freely. Relapse prevention is recommended as the last module. Study participants are asked to work on approximately one to two modules per week. The time required to finish one module may vary, but will usually not exceed 60 min. The intervention is self-paced, so that participants are able to work on topics they prioritize, such as emotional issues rather than positive symptomatology [73].

**Table 1:** Modules of the online program EviBaS

Name	Description
Introduction	Reflect on thoughts, feelings and behavior to understand and decrease the symptom burden.
Feelings of Threat	Review the effects of paranoia on individual goals and needs.
Voice Hearing	Learn strategies to reduce distress caused by hearing voices (better coping, influence the evaluation of voices).
Self-Worth	Find forgotten strengths and train a balanced sense of self.
Overcoming Depression	Set up activities and scrutinize depressing thoughts.
Worrying	Minimize upholding factors of worry and tackle worries with problem solving skills.
Sleep	Discuss maintaining factors of the sleep disorder, such as sleep hindering thoughts or disadvantageous surrounding factors.
Mindfulness	Exercise to direct your attention on one thing without judgement.
Metacognition	Learn in interactive exercises to avoid jumping to conclusions and overconfidence in errors.
Social Competence	Plan and train three different types of social situations: enforcing interests, shaping relationships, and winning sympathies.
Relapse Prevention	Collect individual warning signs and plan ahead.

While working with the program, participants will be in contact with a personal moderator if they want to. The moderator will guide the participants through the program with at least one message per week. The main goal of this steady contact is to help participants structure their usage of the program and to encourage regular participation [47]. If necessary (in case of a participant not using the intervention for seven days), the moderator reminds the participant to interact with the program. Participants’ questions are answered within three workdays by moderators. There is a biweekly supervision of all the moderators in the study team led by a licenced psychotherapist with extensive experience in CBTp.

### Measures

The primary outcome is the reduction of psychotic symptoms (positive symptoms such as voice hearing and paranoid delusions) at post assessment (directly after the completion of the intervention). Secondary outcomes include the level of symptomatology at follow-up, the number of dropouts and the results of all secondary questionnaires which evaluate quality of life, depression severity, treatment satisfaction, the influence of treatment expectancy, and process measures, among others (see Table 2). Assessments will be completed at baseline, eight weeks and 32 weeks. For an overview of all primary and secondary outcome measures, predictors and moderators, as well as process measures, see Table 2.

**Table 2:** An internet-based intervention for people with psychosis (EviBaS): Schedule of enrolment, intervention and assessments

	Study period				
	Enrolment	Allocation	Post-allocation		Close-out
Timepoint	t-1	t0	t1 intervention/waiting period	t2 post assessment	t3 follow-up assessment
Eligibility screen	X				
Informed consent	X				
Allocation		X			
Interventions:					
EviBaS intervention group			X		
Waiting list control group			X		
Assessments:					
Primary outcome measures					
Positive and Negative Syndrome Scale (PANSS)	X			X	X
Mini International Neuropsychiatric Interview (MINI)	X			X	X
Paranoia Checklist	X			X	X
Launay–Slade Hallucination Scale (LSHS-R)	X			X	X
Secondary outcome measures					
Delusion and Voices Self-Assessment (DV-SA)	X			X	X
Rosenberg Self-Esteem Scale (RSES)	X			X	X
Insomnia Severity Index (ISI)	X			X	X
Penn State Worry Questionnaire - Abbreviated (PSWQ-A)	X			X	X
Patient Health Questionnaire (PHQ-9)	X			X	X
Box Task	X			X	X
Mindful Attention Awareness Scale (MAAS)	X			X	X
Interpersonal Competence Questionnaire (ICQ; subscales initiation and negative assertion)	X			X	X
Incongruence questionnaire (K-INK)	X			X	X
World Health Organization Quality of Life Assessment (WHO-QoL-BREF)	X			X	X
Internalized Stigma of Mental Illness – short version (ISMI)	X			X	X
Predictors and moderators					
Medication Adherence Rating Scale (MARS-D)	X			X	X
Attitudes towards Psychological Online Interventions (APOI)	X				
Credibility/Expectancy Questionnaire (CEQ)	X				
University of Rhode Island Change Assessment (URICA)	X				
Client Satisfaction Questionnaire (CSQ)				X	
Questionnaire Side Effects Psychosis and Internet (QueSPI)				X	
Process measures					
Working Alliance Inventory – Short Revised (WAI-SR)		X			
Intermediate Assessments Questionnaire		X			

### Primary outcome measures

#### Positive and Negative Syndrome Scale (PANSS)

The PANSS [71] was the first standardized evaluation tool for symptoms of schizophrenia [74]. It assesses 30 symptoms, which can be grouped into five factors: positive symptoms, negative symptoms, disorganization, excitement, and emotional distress [1]. The positive symptom factor serves as the primary outcome of this study. It includes nine items (delusions, hallucinations, unusual thought content, suspiciousness, grandiosity, somatic concern, active social avoidance, lack of judgment and insight, and (less) difficulty in abstraction). In this study, clinicians administer the PANSS via telephone.

#### MINI International Neuropsychiatric Interview (MINI)

The MINI is a diagnostic structured interview for the assessment of psychiatric diagnoses [70], with a corresponding German version [75]. The specificity of the MINI was reported as good for all diagnoses (ranging from 0.72 to 0.97) [70]. In this study, a part of the MINI (depressive episode, suicidality, manic episode, and psychosis) was also administered via telephone.

#### Paranoia Checklist

The Paranoia Checklist was developed by Freeman et al. in 2005 [76] and assesses the frequency, degree of conviction, and associated distress of a wide range of paranoid thoughts. The three subscales each include the same 18 items, which are rated on a five-point Likert scale: The first subscale measures the frequency of paranoid thoughts (ranging from ‘does not apply at all’ to ‘applies very well’; adapted), the second subscale measures the degree of conviction (from ‘not at all convinced’ to ‘absolutely convinced’) and the third subscale surveys the level of distress (from ‘not distressing’ to ‘very distressing’). Cronbach’s alpha, as an estimate of reliability, is reported as .90 or above, which stands for an excellent internal consistency of the Paranoia Checklist [76].

#### Launay–Slade Hallucination Scale (LSHS-R)

The LSHS-R is the revised version of the LSHS, developed by Launay and Slade in 1981 [77]. It includes 12 items and measures the predisposition to hallucinations on a wide spectrum [78]. There is a German version of the LSHS-R, which shows a Cronbach’s alpha of .87 in a patient sample and therefore is comparable to the original [79]. The five-point Likert scale ranges from 0 (‘certainly does not apply to me’) to 4 (‘certainly applies to me’) and there is a sum score that will be compared between both groups of participants.

### Secondary outcome measures

#### Delusion and Voices Self-Assessment (DV-SA)

The DV-SA has two subscales, a Delusions Scale (DS) and a Voices Scale (VS). The former contains five items to assess patients' opinions about the dominant delusional idea and the latter includes ten items about the subjective dimensions of auditory hallucinations [80]. All responses of the participants are rated on two four-point Likert scales, from 0 (absence of problems) to 3 (the severest of problems), with the highest achievable total score of 15 for the DS and 33 for the VS [80]. For this study, we changed the time period referenced in the DV-SA from 1 month to 1 week.

#### Incongruence questionnaire (K-INK)

The K-INK [81] is a short version of a questionnaire (INK) that measures the degree of realization of motivational goals in the participant’s life. Those goals can be classified into two groups: approach goals and avoidance goals. On a five-point Likert scale, the K-INK measures approach incongruence, avoidance incongruence, and total incongruence. Internal consistency (as measured by Cronbach’s alpha) for the K-INK is reported as ranging between .52 and .87 [81].

#### World Health Organization Quality of Life Assessment (WHO-QoL-BREF)

The WHO-QoL-BREF consists of 26 items and is a standard questionnaire to measure the quality of life [82]. Cronbach’s alpha values range from .66 to .84, indicating an acceptable internal consistency [82]. The WHO-QoL-BREF demonstrates good discriminant validity [82]. In this study, it is administered both in baseline and post assessments and the participants have to indicate their level of agreement on a five-point rating scale with changing answer formats.

#### Rosenberg Self-Esteem Scale (RSES)

The RSES measures self-esteem on a four-point Likert scale. The RSES shows a high reliability and validity for global self-worth [83]. It demonstrates an excellent internal consistency (*Guttman* scale coefficient of reproducibility of .92) [84]. Moreover, the two week test-retest reliability revealed high correlations of .85 and .88, indicating excellent stability [84]. Higher scores on the RSES indicate higher self-esteem.

#### Insomnia Severity Index (ISI)

The ISI is a brief measure for insomnia and is composed of seven items. Each of these items is rated on a five-point Likert scale, ranging from 0 (‘not at all’) to 4 (‘extremely’). Studies reported adequate psychometric properties for ISI versions in English and French [85, 86].

#### Penn State Worry Questionnaire - Abbreviated (PSWQ-A)

The PSWQ [87] is a questionnaire designed to assess the tendency to worry. In this study, an abbreviated eight-item version (PSWQ-A) was used [88]. The measure is scored on a five-point Likert scale from 1 (‘not at all typical’) to 5 (‘very typical’). The PSWQ-A items have good internal consistency highlighted by reported Cronbach’s alpha ranging from .87 to .89 and .94 [88, 89]. Scores for the PSWQ-A range from 8 to 40 [90].

#### Patient Health Questionnaire (PHQ-9)

The PHQ-9 measures depression severity [91]. It scores all nine DSM-IV criteria for depression on a rating scale from 0 (‘not at all’) to 3 (‘nearly every day’). A score of 20 represents severe depression. Internal consistency of the PHQ-9 is excellent with a Cronbach’s alpha of between 0.86 and 0.89 [91]. In this study, the short German version (PHQ-D) was used [92] and the item on suicidality also serves as an indication of the necessity of exclusion from the study.

#### Box Task

In the Box Task [93], participants are confronted with grey boxes on the computer screen, concealing two distinct colours. Participants have to gather information about which of the two colours is more frequent by clicking on said boxes. When they decide that they have gathered sufficient information, they can choose the more frequent colour. This experimental paradigm has been administered in a previous study [94]. If the amount of information an individual gathers before making a decision is low this indicates a tendency to jump to conclusions [27]. Jumping to conclusions has been found to be associated with schizophrenia and delusions [95].

#### Mindful Attention Awareness Scale (MAAS)

The MAAS is a questionnaire measuring mindfulness on a six-point Likert scale. Cronbach’s alpha of the MAAS has been reported as .81 [96].

#### Interpersonal Competence Questionnaire (ICQ)

The ICQ is originally a 40-item questionnaire for the assessment of five domains of interpersonal competence [97]. In this study, only two of the five domains are surveyed: initiation of relationships and negative assertion. Moreover, a recently published short version of the ICQ (called ICQ-15) [98] was used to pick out the six items of the two subscales (three for each domain). The internal consistency of the total scale was high (Cronbach’s alpha = .87) and the reliability coefficients of the subscales were the highest two coefficients of the five subscales: .73 (for initiation of relationships) and .75 (for negative assertion) [98]. The German version of this questionnaire has been validated as well [99].

#### Internalized Stigma of Mental Illness (ISMI) – Short version

The ISMI is a questionnaire measuring the internalized stigma of participants [100]. In this study, the short version of the ISMI was used, which includes 10 items with four-point Likert scales [101]. To evaluate the results, one calculates the mean of those items. A mean score between 1.00 and 2.50 points stands for no internalized stigma, while a mean score between 2.51 and 4.00 stands for high internalized stigma. The German version of the ISMI showed a high internal consistency (Cronbach’s alpha = .92), which was calculated in a study with 139 participants [102].

### Predictors and moderators

#### Medication Adherence Rating Scale (MARS-D)

The MARS [103] consists of five items rated on a five-point Likert scale. These items measure a participant's non-adherent behavior from 1 (‘always’) to 5 (‘never’). A higher score indicates higher adherence to the prescribed medication. MARS-D is the German adaption of this questionnaire developed by Mahler et al. [104]. Internal consistency of the MARS-D (Cronbach’s alpha ranging from .60 to .69) was reported as satisfactory and comparable to the original [104].

#### Attitudes towards Psychological Online Interventions (APOI)

The APOI measures patients’ attitudes towards an online intervention [105]. It reveals certain prejudgments of a study participant, which might influence the outcome parameters and the motivation. Sixteen items are displayed and the level of agreement with each item can be indicated on a five-point rating scale (ranging from ‘no agreement’ to ‘total agreement’). A factor analysis of the APOI showed four dimensions: (a) scepticism and perception of risks (b) confidence in effectiveness (c) technologization threat and (d) anonymity benefits. The APOI shows acceptable to good internal consistency and a good content validity is assumed, because the construction of items was done deductively as well as inductively [105].

#### Credibility/Expectancy Questionnaire (CEQ)

The CEQ is an economic scale to measure treatment expectancy and rationale credibility [106]. The dimensions measured by the CEQ can possibly moderate the outcome. In this study, participants rate items according to two dimensions - one dimension is related to thinking and one is related to feeling. On four of six items, the rating scale ranges from ‘no agreement’ to the treatment rationale to ‘total agreement’ on a nine-point rating scale. For the remaining two items, participants can indicate the subjective symptom improvement from 0% to 100% in steps of 10%. The CEQ demonstrated a high internal consistency of between .84 and .85 [106].

#### University of Rhode Island Change Assessment (URICA)

The URICA [107] is a measure of readiness to change. The 32-item URICA consists of four subscales (eight items each) that correspond to four stages of change (precontemplation, contemplation, action and maintenance) [108]. Internal consistency for the total URICA was reported as excellent (Cronbach’s alpha = .83) [109]. This study used a short version of the German URICA (URICA-S) [110], where items are rated on a five-point Likert scale ranging from 1 (‘not applicable at all’) to 5 (‘very applicable’).

#### Client Satisfaction Questionnaire (CSQ)

The CSQ asks participants to what extent they were satisfied with the intervention [111]. The internal consistency of the CSQ was .93, which stands for an excellent score. There is evidence for a strong construct validity of the CSQ as well [111]. Because it measures treatment satisfaction, it can only be administered at post assessment. Due to its shortness and comprehensiveness, the CSQ is very suitable for mailed surveys [112] and can therefore also be administrated in an online format. The German version (called ZUF-8) [113] is being used in this current study. The eight items can be answered on a four-point rating scale [113].

#### Questionnaire about Side Effects Psychosis and Internet (QueSPI)

This questionnaire assesses the negative effects of internet-based interventions for psychotic patients. It was developed within the research group as part of a pilot study leading up to the current project [114]. Detailed information can be found in the Additional file 1.

### Process measures

#### Working Alliance Inventory – Short Revised (WAI-SR)

As a measure of the weekly variation of the therapeutic alliance, the WAI-SR [114] was included in the study in its German version [115]. It goes back to the Working Alliance Inventory (WAI) [116] and differentiates between goal, task and bond alliance dimensions. Each of those dimensions is represented by four items. The WAI-SR uses a five-point rating scale representing the frequency of positive alliance experiences (ranging from ‘rarely’ to ‘always’). Cronbach’s alpha of the subscale scores ranges from .85 to .90, and of the total scores from .91 to .92, indicating excellent internal consistency [114].

#### Intermediate Assessments Questionnaire

The study group designed a questionnaire to briefly measure symptoms and mental states that were expected to change over the course of the online intervention. Each therapeutic topic covered in the online intervention is represented by a single item in the intermediate assessments questionnaire, such as auditory hallucinations, quality of sleep, self-worth, worry, or depression. Additionally, potentially psychosis-related thinking styles (e.g. jumping to conclusions) are assessed via single items. Five out of 14 items were newly created by the authors, the remaining 9 items were adopted from German versions of established questionnaires (e.g. PHQ-9) [117], or taken and translated from experience sampling studies on schizophrenia (e.g. ‘I feel suspicious‘) [118]. The intermediate assessments questionnaire uses a five-point rating scale ranging from ‘not at all true’ to ‘absolutely true’ and can be read in the Additional file 2.

### Data collection and management

At baseline, participants complete an online assessment including several questionnaires described above. An electronic informed consent and demographic questions will be displayed at the beginning of this online survey. Participants must also indicate their e-mail address and telephone number, which are processed independently from other data. The process of baseline assessment lasts approximately 35 to 40 min. After completion and if no inclusion criteria is not met, a telephone interview with the participant will be arranged. This telephone interview includes two diagnostic interviews (MINI and PANSS, also described above) and the development of an individual emergency plan in case of acute suicidality or psychotic relapse with each participant. The interview lasts approximately 45 to 60 min. If the participant scores below the cutoff on all of the PANSS items (delusions, hallucinations or suspiciousness/persecution), or if the participant reports neither current nor past psychotic symptoms, he or she is excluded from participation and receives a short self-help manual as a compensation for the assessment participation. When inclusion criteria are met, the participants are randomized to one of two groups. The participant either gets access to the online intervention program immediately or after a waiting period of eight weeks. After completion of the intervention period, post assessment and a second telephone interview are administered. The same procedure takes place during the follow-up assessment six months after the intervention period.

### Statistical analyses

Based on the intention to treat sample, a linear mixed-model repeated measures ANOVA with time (T1-T2) as a within-group factor and study condition as a between-group factor will be used for the main research question. Mixed-model repeated measures ANOVA uses all available data of each subject and does not require the substitution of missing values. Sensitivity analysis will be conducted to analyze the impact of dropout on the results. The significance level is set at 5%. We are also interested in possible mediators and/or moderators of the relation between the internet-based self-help intervention and positive psychotic symptoms. We will therefore test whether predictors identified in the literature, such as treatment motivation, working alliance or usage of the intervention could mediate and/or moderate the main effect [119]. To evaluate these possible predictors of treatment outcome, we use change scores of outcome measures as the dependent variables.

### Ethical aspects and data safety

The *Cantonal Ethics Committee Bern* (ID: 03/14) as well as the *German Society for Psychology* (ID: SM052015_CH) have approved of this study. Data safety is ensured by several means: The program and app usage are independent of any personal data. Conversely, the communication via the secured e-mail system contains no information that would allow the identification of a participant in EviBaS. Sensitive data (where personal information such as the e-mail address can be linked to login data for the online program) is stored exclusively non-electronically in a locked closet at one study site. Diagnostic staff will not know the identification of the participants in the program and will be blinded for the allocation of participants in the two groups, whereas moderating staff will not know the contact information of the participants. Breach of blinding will be reported. Network security is achieved through SSL encryption. All staff members who are in contact with study participants, are required to fill out a non-disclosure agreement.

## Discussion

EviBaS has been developed as one of the first fully encompassing iCBT programs for people with psychosis. People with schizophrenia spectrum disorders suffer from a heavy burden of symptoms and stigmatization [13]. Psychological evidence-based treatments for schizophrenia exist, but only a small portion of affected people receive them [120]. Bridging the treatment gap in the psychological care for people with severe mental disorders is therefore of utmost importance. This project wants to reach people who do not receive psychological treatment but are looking for support. Internet-based interventions might even be able to reach a group of patients that discontinued a previous face-to-face therapy [121]. Given the efficacy of CBTp on hallucinations and delusions [122], as well as of iCBT in other mental disorders [46], the EviBaS self-help program is expected to reduce symptomatology. Negative effects and long-lasting effects of the treatment will be assessed. This study will also add to our understanding of how people with schizophrenia use internet-based interventions.

## Additional files

Additional file 1: Questionnaire about Side Effects Psychosis and Internet (QueSPI). (DOCX 20 kb)

Additional file 2: Intermediate Assessments Questionnaire. (DOCX 12 kb)

## Abbreviations

ACT: Acceptance and Commitment Therapy
APOI: Attitudes towards Psychological Online Interventions Questionnaire
CBT: Cognitive-behavioral therapy
CBTp: Cognitive behavioral therapy for psychosis
CEQ: Credibility/Expectancy Questionnaire
CSQ: Client Satisfaction Questionnaire
DV-SA: Delusion and Voices Self-Assessment
EviBaS: Evidence-based self-help intervention
iCBT: Internet-based cognitive behavioral therapy
iCBTp: Internet-based treatments for people with psychosis
ICQ: Interpersonal Competence Questionnaire
ISI: Insomnia Severity Index
ISMI: Internalized Stigma of Mental Illness Questionnaire
K-INK: Incongruence Questionnaire (short version)
LSHS-R: Launay–Slade Hallucination Scale
MAAS: Mindful Attention Awareness Scale
MARS-D: Medication Adherence Rating Scale
MCT: Metacognitive Training
MINI: Mini International Neuropsychiatric Interview
PANSS: Positive and Negative Syndrome Scale
PHQ-9: Patient Health Questionnaire with 9 Items
PSWQ-A: Penn State Worry Questionnaire - Abbreviated
QueSPI: Questionnaire about Side Effects Psychosis and Internet
RCT: Randomized controlled trial
RSES: Rosenberg Self-Esteem Scale
URICA: University of Rhode Island Change Assessment
WAI-SR: Working Alliance Inventory – Short Revised
WHO-QoL-BREF: World Health Organization Quality of Life Assessment
