# Supplementary material for: An internet-based intervention for people with psychosis (EviBaS): study protocol for a randomized controlled trial
Source: BMC Psychiatry. 2018 Apr 13;18:102. doi: 10.1186/s12888-018-1644-8 (PMC5899332; doi:10.1186/s12888-018-1644-8)
Supplement: Supplementary file 1 — Questionnaire about Side Effects Psychosis and Internet (QueSPI). (DOCX 20 kb) [file 12888_2018_1644_MOESM1_ESM.docx]

**Additional file 1**

**Questionnaire about Side Effects Psychosis and Internet (QueSPI)**

This questionnaire was developed by members of our research group. For the current purpose, it was translated from German. The ratings are rated on a four-point Likert scale ranging from ‘I do not agree at all’ to ‘I agree completely’.

1. The self-help program has triggered me to lose faith in psychotherapy in general.
2. The self-help program makes me feel like I am responsible for my problems.
3. The program was not what it pretended to be.
4. Participation in the self-help program reduced my interest to participate in a psychotherapy with personal contact.
5. The self-help program did not sufficiently address my needs.
6. Because of the self-help program I plan to discontinue to take my medications or I already stopped taking them.
7. I experienced my participation in the self-help program as a personal failure.
8. The self-help program makes me feel abnormal.
9. I felt the program spied on me.
10. I often did not understand what the self-help program tried to tell me.
11. The self-help program dictated how I have to think.
12. I missed human interaction in the self-help program.
13. I worried about data safety in the self-help program.
14. The self-help program overwhelmed me with its abundance of information.
15. I feared that the self-help program could increase my symptoms.
16. I felt left alone by the self-help program.
17. The moderator often misunderstood me.
18. My hope of improvement due to the self-help program was disappointed.
19. The self-help program contained hidden messages.
20. The self-help program was a secret test.
21. I experienced technical difficulties that bothered me.
22. The recommendations from the self-help program were different from those of my doctor/psychiatrist/psychotherapist.
23. My moderator did not respond fast enough to my urgent requests.
24. To trust others is harder for me due to the self-help program.
25. The usage of the app or the worksheets have put pressure on me.
26. I feel ashamed, sad or angry because I did not exercise enough in my everyday life.
27. I am afraid that someone might have noticed that I used the self-help program or the app.
28. The usage of the program caused me to have more conflicts with others.
    1. Have new symptoms emerged during the self-help intervention period? If yes, which ones? Do you think those new symptoms emerged because of the self-help program?
    2. Did some symptoms get worse during the self-help intervention period? If yes, which ones? Do you think that this is because of the self-help program?
    3. Were there certain events which lead to a worsening of symptoms during the self-help intervention period? If yes, which ones? Are those events in any relation to the self-help program?
