# Supplementary material for: An internet-based intervention for people with psychosis (EviBaS): study protocol for a randomized controlled trial
Source: BMC Psychiatry. 2018 Apr 13;18:102. doi: 10.1186/s12888-018-1644-8 (PMC5899332; doi:10.1186/s12888-018-1644-8)
Supplement: Supplementary file 2 — Intermediate Assessments Questionnaire. (DOCX 12 kb) [file 12888_2018_1644_MOESM2_ESM.docx]

**Additional file 2**

**Intermediate Assessments Questionnaire**

This questionnaire was developed by members of our research group. For the current purpose, it was translated from German. The ratings are rated on a five-point Likert scale ranging from ‘does not apply at all’ to ‘applies completely’.

1. I am suspicious.

2. I hear voices that are not audible for others.

3. If I am sure of a thing, then it is also true.

4. I am content with myself.

5. My worries are becoming too much to handle.

6. I can challenge how I evaluate a situation.

7. I take as much information as possible into consideration before making a decision.

8. I feel depressed, melancholic or helpless.

9. I feel anxious.

10. I feel happy.

11. I concentrate only on what I currently do and on nothing else.

12. If I had to conduct a conversation with a new acquaintance, who I wanted to get to know more, I would succeed.

13. I would currently qualify my quality of sleep as ‚good‘.

14. In the past 24 hours, something very stressful happened to me.
